# Supplementary material for: Development of Ic-ELISA and Colloidal Gold Lateral Flow Immunoassay for the Determination of Cypermethrin in Agricultural Samples
Source: Biosensors (Basel). 2022 Nov 21;12(11):1058. doi: 10.3390/bios12111058 (PMC9688530; doi:10.3390/bios12111058)
Supplement: Supplementary file 1 [file biosensors-12-01058-s001.zip › biosensors-1983723-supplementary.pdf]

biosensors

MDPI

Supplementary Material

Development of Ic-ELISA and Colloidal Gold Lateral Flow Immunoassay for the Determination of Cypermethrin in Agricultural Samples

Lianrun Huang [1,2], Fuxuan Zhang [1,2], Fangxuan Li [1,2], Yin Jia [1,2], Minghua Wang [1,2], Xiude Hua [1,2,*] and Limin Wang [1,*]

[1] College of Plant Protection, Nanjing Agricultural University, Nanjing 210095, China
[2] State and Local Joint Engineering Research Center of Green Pesticide Invention and Application, Nanjing 210095, China
* Correspondence: huaxiude@njau.edu.cn (X.H.); wlm@njau.edu.cn (L.W.)

Part S1. The checkerboard procedure of ic-ELISA

To optimize the concentrations of the coated antigen and antibody of ic-ELISA, checkerboard titration was carried out. Briefly, the coating antigen was diluted into a series of concentrations (2, 1, 0.5, 0.25 and 0.125 μg/mL) and coated five row wells of microplates. After the plates were blocked, the antibody was diluted into serial concentrations (2, 1, 0.5, 0.25 and 0.125 μg/mL) and added into the coated wells column by column. The following steps were the same as those used in ic-ELISA procedure. The concentration of antibody and coating antigen of the well, which of the OD value is about 1.0, are determined as the optimal working condition.

Table S1. The hapten-to-protein molar ratios of conjugates.

Conjugates	Coupling ratio	Conjugates	Coupling ratio
H1-KLH	12.1:1	H4-BSA	17.1:1
H1-BSA	3.6:1	H5-BSA	1.3:1
H2-BSA	14.9:1	H6-BSA	6.5:1
H3-BSA	2.6:1	H7-BSA	2.0:1

Table S2. Sensitivities of anti-CYP antibodies using hapten 1 as coating antigen.

Compound	Cell line	IC_{50} (ng/mL)
Cypermethrin	2G7H9	19
	2G9A8	176
	1E7D5	314
	6F1G10	575

Table S3. Effect of of sodium ion concentration, pH, type and content of organic solvents on sensitivity of ic-ELISA for determination of CYP.

Working buffer conditions		A_{max}	IC_{50} (ng/mL)	A_{max}/IC_{50}
**10% Methanol**		1.57	7.67	0.20
10% Acetonitrile		1.15	8.88	0.13
10% Acetone		1.25	7.99	0.17
10% Dimethyl sulfoxide		1.38	11.03	0.13
Methanol	5%	1.51	30.63	0.05
	10%	1.37	8.60	0.17
	20%	0.99	20.59	0.05

	40%	0.65	24.47	0.03
Na$^+$ (mol/L)	0.07	2.40	43.31	0.06
	0.14	2.31	9.22	0.25
	0.2	1.80	13.58	0.13
	0.3	1.32	2.42	0.55
	0.4	0.65	2.94	0.22
pH	6	0.81	4.41	0.18
	7	1.17	5.24	0.22
	7.4	1.24	3.09	0.40
	8	1.10	8.06	0.14
	9	0.70	4.03	0.17

Table S4. Influence of sodium ion concentration, pH, type and content of organic solvents on sensitivity of LFIA for determination of CYP.

**Working buffer conditions**			0 μg/mL	0.3 μg/mL	0.4 μg/mL	0.6 μg/mL
**Na$^+$ (mol/L)**		**0.07**	+++[a]	+++	++–	–––
		0.14	+++	+++	++–	+––
		0.25	+++	+++	++–	+––
		0.5	++–	++–	++–	+––
		1	++–	++–	++–	+––
		2	+––	+––	+––	–––
pH		6	+++	+++	++–	–––
		7	+++	+++	+––	–––
		7.4	+++	+++	++–	–––
		8	++–	++–	+––	–––
		9	+––	+––	–––	–––
Organic solvents (v%)	Methanol	10	+++	+++	+––	–––
		20	+++	+++	+––	+––
		30	++–	++–	+––	+––
		40	+––	+––	+––	–––
	Acetonitrile	10	+++	+++	++–	+––
		20	++–	++–	+––	–––
		30	+––	+––	+––	–––
		40	–––	–––	–––	–––
	Acetone	10	+++	+++	++–	+––
		20	+++	+++	+––	+––
		30	++–	++–	+––	–––
		40	+––	+––	–––	–––
	Dimethyl sulfoxide	10	+++	++–	+––	–––
		20	+++	+++	+––	–––
		30	++–	++–	+––	–––
		40	++–	++–	+––	–––

a: More plus signs (+) mean darker red; --- means no color band.

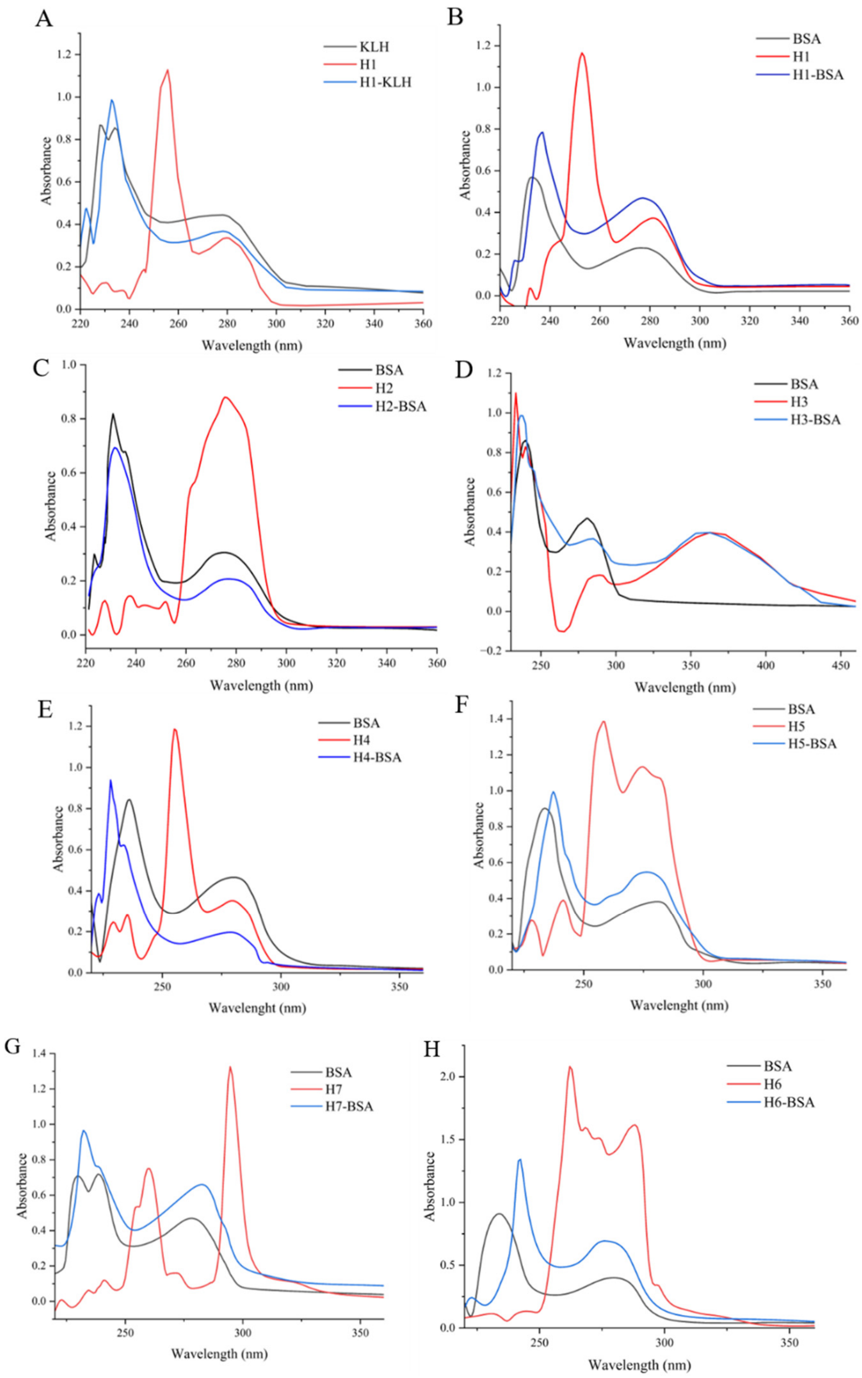

Figure S1. The UV-Vis spectra of hapten-carrier protein conjugates. (A) Hapten 1-KLH; (B) Hapten 1-BSA; (C) Hapten 2-BSA; (D) Hapten 3-BSA; (E) Hapten 4-BSA; (F) Hapten 5-BSA; (G) Hapten 6-BSA; (H) Hapten 7-BSA.

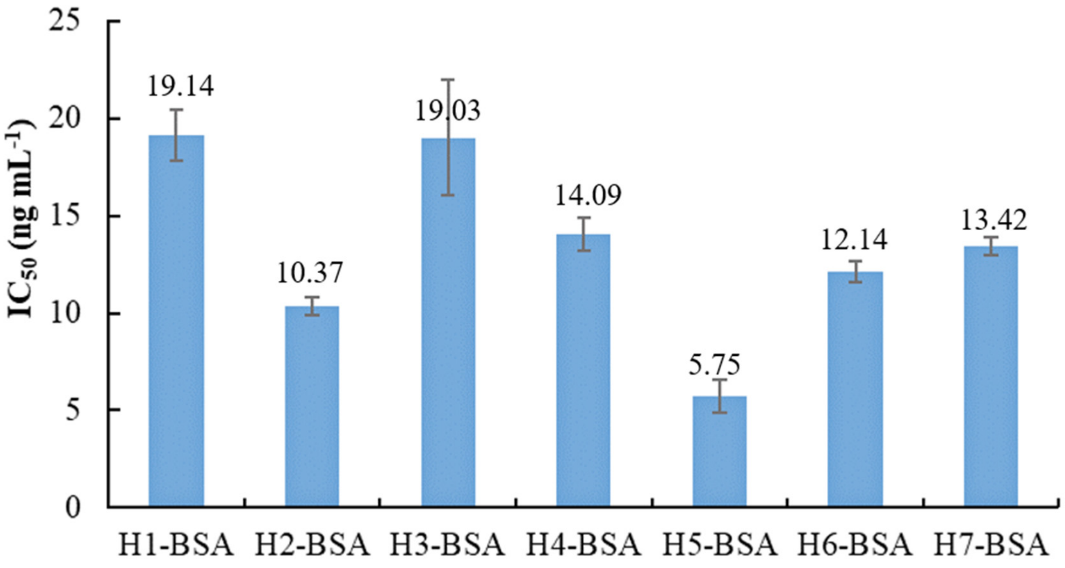

Figure S2. The sensitivity of mAb 2G7H9 using different coating antigens.

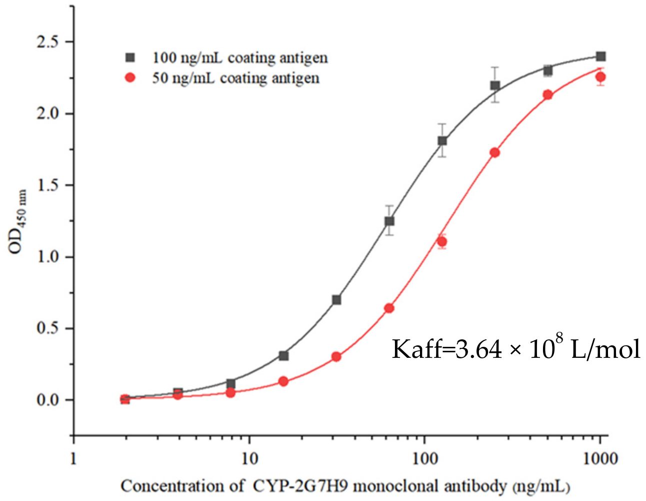

Figure S3. The Kaff value of mAb 2G7H9.

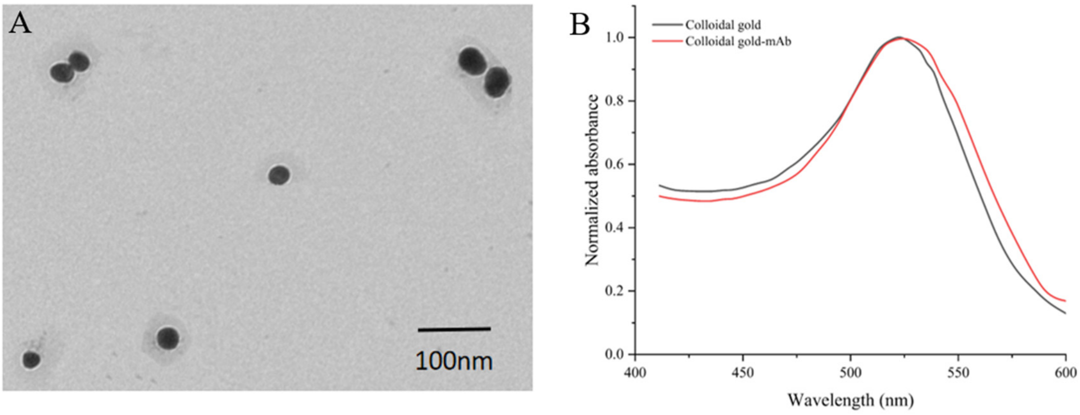

Figure S4. Identification of colloidal gold and colloidal gold-mAb. (A) The transmission electron micrograph of the colloidal gold; (B) The UV-Vis spectra of colloidal gold and colloidal gold-mAb.

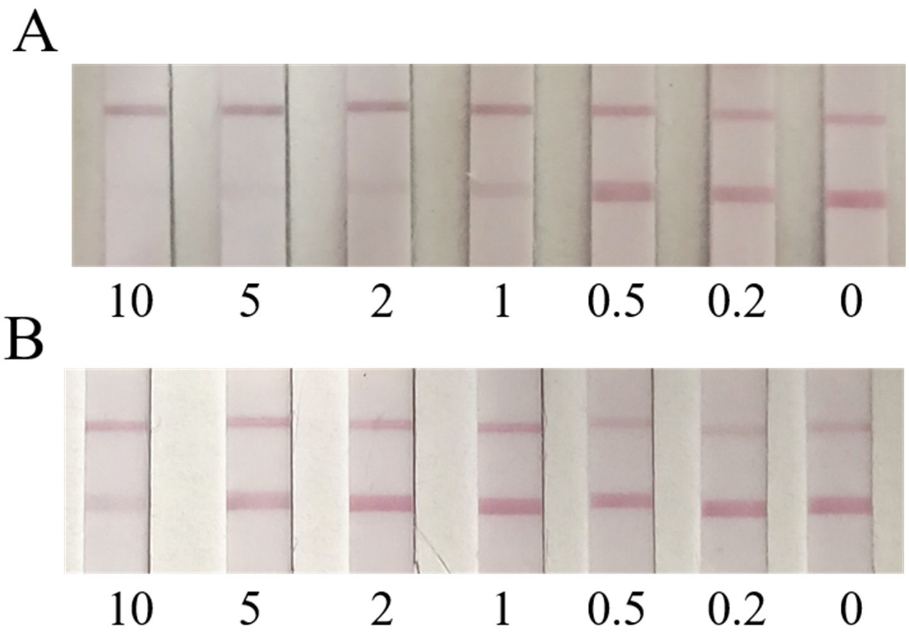

Figure S5. The vLOD of LFIA for cyfluthrin (A) and cyhalothrin (B) determination, the concentrations from left to right were 10, 5, 2, 1, 0.5, 0.2 and 0 μg/mL.
